# Extracapsular Enucleation Versus Partial Superficial Parotidectomy and Extracapsular Dissection in Warthin’s Tumor: A Retrospective Matched Cohort Study

**DOI:** 10.3390/jcm15083026

**Published:** 2026-04-15

**Authors:** Michael Kostares, Evangelos Kostares, Maria Kakazani, Marina Karaiskou, Matilda Chatzipanagiotou, Maria Kantzanou, Paul Stampouloglou, Maria Piagkou, Spiridon Laskaris

**Affiliations:** 1 Department of Anatomy, National and Kapodistrian University of Athens, 106 79 Athens, Greece; mapian@med.uoa.gr; 2 Department of Otorhinolaryngology—Head and Neck Surgery, “Metaxa” Memorial Anticancer Hospital, 185 37 Piraeus, Greece; 3 Department of Oral and Maxillofacial Surgery, National and Kapodistrian University of Athens, “Evangelismos” General Hospital, 106 79 Athens, Greece; 4 Department of Microbiology, Medical School, National and Kapodistrian University of Athens, 106 79 Athens, Greece; mkatzan@med.uoa.gr

**Keywords:** Warthin’s tumor, extracapsular enucleation, extracapsular dissection, partial superficial parotidectomy, postoperative complications

## Abstract

**Background/Objectives:** Warthin’s tumor (WT) is a benign parotid neoplasm increasingly managed with tissue-preserving surgical approaches to reduce postoperative morbidity. Partial superficial parotidectomy (PSP) and extracapsular dissection (ECD) are commonly performed, whereas extracapsular enucleation (EN) remains less systematically evaluated. This study compared postoperative morbidity among EN, PSP, and ECD in a matched cohort of patients with unifocal WT. **Methods:** A retrospective matched cohort study was conducted at a single tertiary referral center, including patients with histologically confirmed, unifocal WT treated between 2009 and 2023. A total of 360 patients were organized into 120 matched triplets (EN, PSP, ECD), with exact matching on age group and sex and balancing for smoking status, body mass index, and alcohol use. To enable comparison under technically uncomplicated conditions, cases with documented intraoperative capsular rupture or tumor spillage were excluded. The primary endpoint was overall postoperative morbidity, defined as the occurrence of at least one predefined complication. Associations between surgical technique and morbidity were assessed using conditional logistic regression, with estimation of odds ratios (ORs), absolute risk differences (RDs), and numbers needed to treat (NNT). **Results:** Overall complication rates were 8.3% after EN, 29.2% after ECD, and 32.5% after PSP (*p* < 0.001). EN was associated with lower odds of postoperative complications compared with ECD (OR 0.23, 95% CI 0.10–0.50) and PSP (OR 0.18, 95% CI 0.08–0.41). Adjusted absolute risks were 8.3% for EN, 29.3% for ECD, and 32.4% for PSP, corresponding to absolute risk differences of 21% and 24% and numbers needed to treat of 4.8 and 4.2, respectively. **Conclusions:** In this selected cohort of unifocal, anatomically favorable Warthin’s tumors without intraoperative capsular violation, ΕΝ was associated with lower observed postoperative morbidity compared with ECD and PSP. These findings are context-dependent and reflect outcomes achieved under strict selection and technical conditions. Therefore, they should not be extrapolated to anatomically complex, inflamed, or diagnostically uncertain lesions. Prospective multicenter studies with standardized selection criteria are warranted to better define the role of EN in contemporary WT management.

## 1. Introduction

Warthin’s tumor is one of the most common benign neoplasms of the parotid gland, predominantly affecting older individuals and exhibiting a strong association with tobacco exposure [1]. The tumor is characterized by a typically indolent biological behavior, with malignant transformation being rare [2]. Warthin’s tumor may present as unilateral or bilateral disease and is known for its tendency toward multifocality within the parotid gland. These biological and clinical features have shaped contemporary management strategies, which increasingly prioritize functional preservation and minimization of treatment-related morbidity alongside diagnostic certainty and symptom control [3].

Historically, superficial parotidectomy was widely adopted as the standard surgical approach for benign parotid tumors, largely extrapolated from management principles established for pleomorphic adenoma [4]. While superficial parotidectomy provides reliable tumor control, its association with facial nerve dysfunction and sensory disturbances prompted exploration of less extensive surgical approaches [5,6,7]. This evolution reflects changing priorities in benign parotid surgery rather than a reassessment of oncologic adequacy [8,9].

In this context, more conservative surgical techniques were introduced with the aim of limiting the extent of resection while preserving facial nerve function and parotid tissue. Partial superficial parotidectomy involves identification of the facial nerve trunk and excision confined to the tumor-bearing portion of the superficial lobe [10]. Extracapsular dissection, by contrast, consists of removal of the lesion with a narrow margin of surrounding parotid tissue without planned exposure of the main facial nerve trunk, although peripheral nerve branches may be encountered during dissection [11]. Extracapsular enucleation represents the most limited approach and is defined as dissection directly along the tumor capsule without removal of a surrounding parenchymal cuff.

As the extent of resection decreases, accurate case selection and diagnostic certainty become increasingly important. Optimizing conservative surgery requires robust preoperative characterization [3,12]. Multidisciplinary algorithms that integrate FNAC with clinical and ultrasonographic features improve diagnostic confidence when labeling a parotid mass as Warthin’s tumor and thereby de-risk selection of extracapsular enucleation or extracapsular dissection [13,14,15]. Equally, contemporary decision frameworks acknowledge patient-tailored care: in older, comorbid, asymptomatic patients with classic Warthin’s tumor, active surveillance is increasingly chosen, reserving intervention for growth, symptoms, cosmetic concerns, or diagnostic ambiguity [16,17].

Beyond conventional open approaches, several innovations reflect a global trajectory toward minimal morbidity in benign salivary surgery. Emerging literature explores endoscopic/robotic parotidectomy to conceal scars and potentially reduce soft-tissue morbidity [18,19]. In parallel, non-surgical modalities such as radiofrequency ablation are under review as alternatives for poor surgical candidates or for patients prioritizing cosmesis/rapid recovery; early evidence suggests feasibility and symptom relief, though long-term control data remain limited [20,21,22].

Based on the biological characteristics of Warthin’s tumor and the availability of surgical approaches with differing degrees of tissue preservation, it is clinically relevant to consider whether postoperative morbidity profiles vary among partial superficial parotidectomy, extracapsular dissection and extracapsular enucleation. Therefore, the primary aim of this study is to compare postoperative complication rates among partial superficial parotidectomy, extracapsular dissection and extracapsular enucleation for histologically confirmed Warthin’s tumor in a matched cohort. Secondary aims are to quantify absolute risk differences and numbers-needed-to-treat, and to explore effect modification by potential confounders.

## 2. Materials and Methods

### 2.1. Study Design and Eligibility Criteria

This retrospective matched cohort study was based on the surgical database of the ENT Clinic of the “Metaxa” Memorial Anticancer Hospital in Piraeus, Greece. The study included parotid gland operations performed between January 2009 and December 2023. All procedures were carried out by experienced surgeons within the same departmental ENT surgical team.

Within this surgical cohort, only cases with histologically confirmed Warthin’s tumor were considered eligible for inclusion. Inclusion criteria comprised (i) unifocal disease without evidence of multifocality or bilaterality, (ii) absence of prior parotid surgery, and (iii) availability of perioperative and follow-up documentation sufficient to ascertain predefined postoperative outcomes and matching variables. Patients with other benign or malignant histologies, multifocal or recurrent Warthin’s tumor, incomplete records for matching variables or postoperative complications, or documented intraoperative capsular rupture or tumor spillage were excluded prior to matching. This exclusion was applied across all surgical techniques to allow comparison of postoperative morbidity following technically successful resections, acknowledging that capsular rupture represents a distinct intraoperative event influenced by tumor characteristics and operative complexity. The study selection and matching process is summarized in Supplementary Materials (Figure S1).

### 2.2. Preoperative Assessment and Surgical Technique Selection

Preoperative evaluation was performed in all patients and aimed at confirming the benign nature of the lesion and defining its anatomical characteristics. Ultrasonography (US) was used as the primary imaging modality in all cases, while computed tomography (CT) or magnetic resonance (MRI) imaging was obtained when additional anatomical detail was required [23]. Fine-needle aspiration (FNA) cytology was routinely performed preoperatively to support the diagnosis of benign disease [24]. Facial nerve function was intact in all patients prior to surgery, as assessed using the House-Brackmann grading system [25]. The diagnosis of Warthin’s tumor was subsequently confirmed by histopathological examination of the resected specimens.

Selection of the surgical technique was guided by standard departmental practice and was based primarily on tumor size, depth, and its anatomical relationship to facial nerve branches, as determined by preoperative imaging and intraoperative findings. The intent of this approach was to tailor the extent of resection to tumor characteristics while preserving facial nerve function and parotid tissue whenever feasible. All procedures were performed under identical preoperative preparation, anesthetic management, and postoperative care protocols.

Partial superficial parotidectomy was performed in cases in which tumors were in close proximity to facial nerve branches, such that safe excision required deliberate identification of the nerve. The procedure was carried out through a modified Blair incision, with elevation of skin flaps and identification of the main facial nerve trunk at its emergence from the stylomastoid foramen, followed by dissection of the relevant facial nerve division as dictated by tumor location. Resection was limited to the tumor-bearing portion of the superficial lobe, which was excised en bloc with a cuff of surrounding parotid tissue, while uninvolved facial nerve branches were carefully preserved.

Extracapsular dissection was applied to superficially located, well-circumscribed tumors in which preservation of surrounding parotid tissue was prioritized and formal dissection of the main facial nerve trunk was not considered necessary. After skin flap elevation, the parotid tissue was opened near the lesion, and the tumor was exposed and removed in an extracapsular plane with a narrow rim of adjacent parotid tissue. The main facial nerve trunk was not exposed; however, one or more peripheral facial nerve branches were typically encountered during dissection and were carefully identified and preserved. When a nerve branch was in direct contact with the tumor, limited capsular dissection was performed to allow safe mobilization.

Extracapsular enucleation was reserved for small, well-circumscribed, superficially located tumors with a clearly defined capsule and an evident anatomical separation from facial nerve branches. Tumor removal was achieved through dissection performed directly along the external surface of the tumor capsule, allowing complete mobilization of the lesion without intentional excision of surrounding healthy parotid parenchyma. The main facial nerve trunk was not exposed, and any peripheral branches encountered during capsular dissection were carefully mobilized and preserved.

### 2.3. Outcome Measures and Baseline Variables

Baseline demographic and lifestyle characteristics were documented for all patients preoperatively, including age, sex, smoking status, body mass index (BMI), and alcohol use. Postoperative follow-up focused on surgery-related morbidity. A predefined set of postoperative events was evaluated, including: (i) facial nerve-related complications (facial nerve dysfunction and Frey’s syndrome), (ii) salivary complications (salivary fistula and sialocele or seroma), and (iii) wound-related complications (hematoma requiring evacuation and permanent great auricular nerve sensory dysfunction).

Facial nerve dysfunction was defined as any postoperative weakness persisting beyond 24 h [26]. Recovery was classified as transient when complete resolution occurred within six months and as permanent when dysfunction persisted beyond this interval despite treatment [27]. Frey’s syndrome was identified based on patient-reported gustatory sweating or flushing over the parotid region during mastication or gustatory stimulation, with manifestations recognized more than 30 days after surgery considered late events [28,29]. Salivary fistula was defined as persistent cutaneous salivary leakage following drain removal lasting longer than 48 h or requiring repeated dressing changes [30]. Sialocele or seroma was defined as a clinically evident subcutaneous or subplatysmal fluid collection requiring at least one aspiration [31].

Hematoma was defined as postoperative blood accumulation within the surgical bed that was diagnosed clinically or at surgical re-exploration and required evacuation because of swelling, pain, or risk of flap compromise [32]. Permanent great auricular nerve (GAN) dysfunction was defined as a persistent sensory disturbance—hypoesthesia, anesthesia, or dysesthesia—within the GAN distribution lasting longer than three months postoperatively without evidence of gradual clinical improvement [33]. Sensory assessment was performed using light-touch discrimination with a cotton wisp and pinprick testing over the preauricular and infra-auricular skin corresponding to the GAN territory. Sensory disturbance was recorded when patients reported persistent hypoesthesia, paresthesia, or anesthesia, whereas transient alterations resolving within this interval were not classified as permanent complications. This definition is consistent with previously published criteria for long-term sensory outcomes following parotid surgery [33,34,35].

In addition to individual complications, an overall morbidity measure was derived and considered present when at least one predefined event occurred. A secondary composite measure, consisting of hematoma requiring re-exploration and salivary fistula or sialocele necessitating repeated interventions, was used to capture clinically more severe postoperative morbidity.

Patients were followed for a median of 60 months (interquartile range, 36–108 months), with an early postoperative assessment during the first postoperative week and subsequent evaluations at approximately 6 months, 12 months, and annually thereafter. Follow-up was directed toward postoperative morbidity rather than long-term oncologic outcomes. Tumor recurrence was not evaluated, as surveillance was not uniform across the cohort and retrospective assessment would risk outcome misclassification in a tumor entity characterized by multifocal and bilateral occurrence. In this setting, newly detected postoperative lesions could not be reliably distinguished from metachronous or multifocal disease, and crude recurrence estimates were therefore considered potentially misleading. Although surgical site infection was not predefined as a study outcome, its incidence was documented to provide a comprehensive overview of postoperative safety.

### 2.4. Matching Strategy

The matching protocol was specified a priori and applied after eligibility screening to form comparable groups. Five variables were selected to balance baseline patient risk profiles and lifestyle-related susceptibility to postoperative complications: *age group*, *sex*, *smoking status*, *body mass index (BMI)*, and *alcohol use*. *Age group* was categorized into five strata (<40, 40–49, 50–59, 60–69, and ≥70 years), reflecting the known age distribution of the disease. Smoking status was categorized as never, former, or active smoker. *BMI* was categorized as normal weight, overweight, or obese using WHO thresholds. *Alcohol use* was recorded as none, occasional, or frequent.

A 1:1:1 individual matching protocol was implemented: for each EN case, two controls (one PSP and one ECD) were selected to form a matched triplet. Exact matching was applied for age group and sex, whereas nearest-neighbor matching without replacement was used for smoking status, BMI category, and alcohol use. This resulted in 120 non-overlapping triplets (n = 360). Residual imbalance in the partially matched variables (BMI and alcohol use) was assessed using standardized mean differences (SMD), with values <0.1 considered negligible. Matching was designed to balance baseline patient-related factors associated with postoperative morbidity rather than determinants of surgical technique selection [36]. These variables were selected to reduce confounding related to differential susceptibility to complications, rather than to address confounding by indication [37]. Tumor-specific characteristics that directly guided surgical technique selection—such as size, depth, and inflammatory status—were not uniformly available in the historical record and therefore could not be incorporated into the matching process [38].

### 2.5. Statistical Analysis

All statistical analyses were conducted using Stata/BE 19.5 for Mac (StataCorp LLC, College Station, TX, USA). All categorical variables, including demographic characteristics, lifestyle factors, and postoperative complications, were summarized as absolute counts and percentages. The adequacy of matching across the three surgical groups was assessed for each of the predefined matching variables using Pearson’s χ^2^ test when all expected cell counts were ≥5, and Fisher’s exact test otherwise [39]. All analyses were performed on the final matched analytical cohort, which included only triplets with complete data for the predefined matching variables and postoperative outcomes.

Following descriptive analysis, between-group comparisons of postoperative complication rates were performed for each predefined complication and for the composite endpoint “overall complications.” Pearson’s χ^2^ test was used when expected cell frequencies met minimum assumptions; otherwise, Fisher’s exact test was applied. These unadjusted comparisons provided an initial, clinically interpretable view of complication-specific and overall morbidity differences between surgical techniques.

The primary analytical approach for estimating the association between surgical technique and the composite endpoint was conditional logistic regression stratified by match identifier (triplet ID). This model conditions on each matched triplet, thereby controlling for all covariates that are constant within a match and eliminating confounding by the matching variables [40]. Because age and sex were exactly matched, they are inherently controlled for by design and therefore not included as covariates in the model. Prespecified adjustment was performed only for smoking status, body mass index (BMI) and alcohol use, the partially matched variables. Robust standard errors were clustered at the triplet level. Odds ratios (ORs) with 95% confidence intervals (CIs) were reported.

To provide absolute measures of effect, risk standardization was performed using logistic regression with match fixed effects including surgical technique, smoking status, BMI, and alcohol use. From these models, marginal predicted probabilities of complications were estimated for each surgical technique by averaging over the empirical covariate distribution. Pairwise risk differences (RDs) between EN and each comparator (ECD and PSP) were derived, and the number needed to treat (NNT) was calculated as the reciprocal of the RD, with negative values interpreted as the number needed to harm (NNH) [41]. The uncertainty surrounding RDs and NNTs was quantified via nonparametric cluster bootstrap resampling at the match level (2000 replicates) to preserve the matched data structure; 95% CIs were obtained from the bootstrap distributions [42].

Model adequacy was assessed through standard regression diagnostics, including evaluation of influential matches using leave-one-match-out analyses and assessment of multicollinearity among covariates via variance inflation factors (VIFs) [43,44].

All hypothesis tests were two-sided with a significance threshold of α = 0.05. For secondary and subgroup analyses, *p*-values were interpreted cautiously. Holm’s method was applied as a sensitivity approach for multiple secondary comparisons, without altering the primary inferential framework [45]. Effect estimates are presented with 95% CIs throughout.

The study size was determined by the total number of eligible cases, without a priori sample size calculation [46]. A post hoc power and precision analysis indicated that, with 120 patients per surgical group and the observed baseline complication rates in the non-EN groups, the study had adequate power (>80%) to detect an absolute difference of at least 15% in the composite endpoint between EN and either comparator at a two-sided α level of 0.05, suggesting reasonable precision to detect clinically meaningful differences in morbidity rates [47].

## 3. Results

### 3.1. Baseline Characteristics

A total of 360 patients were included in the analysis. Baseline characteristics are summarized in Table 1. Age and sex distributions were identical across the three groups. Smoking status did not differ between techniques (*p* = 0.914). Body mass index showed a borderline between-group difference (*p* = 0.053), while alcohol consumption differed nominally between techniques (*p* = 0.038). Standardized mean differences indicated good balance for smoking status, with small residual imbalance for BMI and alcohol use.

### 3.2. Overall and Complication-Specific Outcomes

**Figure 1:**
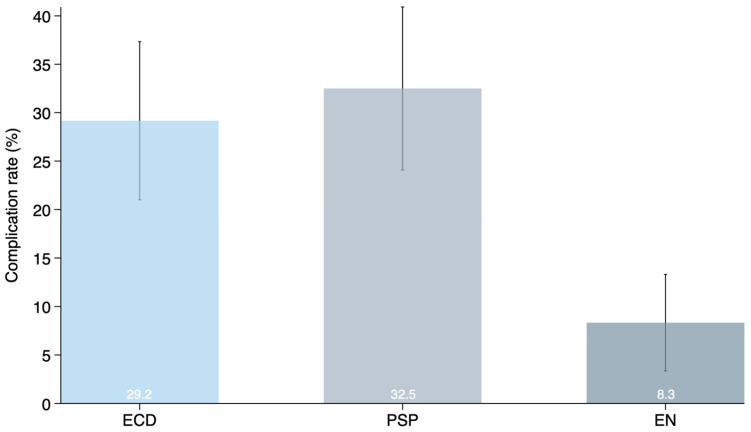
Crude postoperative complication rates after extracapsular enucleation (EN), partial superficial parotidectomy (PSP), and extracapsular dissection (ECD) in patients with Warthin’s tumor. Bars represent 95% confidence intervals (CI) for the crude complication rates. Numerical percentages are shown in white font within the bars for clarity.

Overall postoperative complications occurred in 32.5% after partial superficial parotidectomy, 29.2% of patients treated with extracapsular dissection and 8.3% after extracapsular enucleation, as summarized in Table 2 and Figure 1. Across individual complication categories, extracapsular enucleation was associated with lower observed event rates. Nominal between-group differences were observed for several individual complications; however, none of the individual complication endpoints remained statistically significant after adjustment for multiple comparisons.

### 3.3. Primary and Sensitivity Analyses

Extracapsular enucleation was associated with lower odds of overall postoperative complications compared with both extracapsular dissection (OR 0.23, 95% CI 0.10–0.50) and partial superficial parotidectomy (OR 0.18, 95% CI 0.08–0.41), whereas no statistically significant difference was observed between partial superficial parotidectomy and extracapsular dissection (OR 1.24, 95% CI 0.68–2.28) (Table 3).

Covariate-adjusted absolute risks of postoperative complications were 8.3% for extracapsular enucleation, 29.3% for extracapsular dissection, and 32.4% for partial superficial parotidectomy. These estimates corresponded to absolute risk differences of 21% and 24% for extracapsular dissection and partial superficial parotidectomy, respectively, when compared with extracapsular enucleation, and to numbers needed to treat of 4.8 and 4.2 (Table 4).

Overweight ΒΜΙ was associated with increased odds of postoperative complications (OR 2.37, 95% CI 1.03–5.47). No interaction was observed between surgical technique and either body mass index or alcohol consumption. Across all examined strata, extracapsular enucleation was associated with the lowest predicted probability of postoperative complications.

## 4. Discussion

This study examined postoperative morbidity across three surgical techniques for unifocal Warthin’s tumor within a matched cohort, allowing comparison under controlled patient-related characteristics. Overall complication rates differed between techniques, with higher proportions of patients experiencing at least one postoperative complication after partial superficial parotidectomy and extracapsular dissection, and lower proportions after extracapsular enucleation.

These differences were consistently observed across crude comparisons and covariate-adjusted analyses. No difference in overall postoperative morbidity was identified between extracapsular dissection and partial superficial parotidectomy. Across individual complication categories, lower event rates were generally observed after extracapsular enucleation; however, no single complication endpoint independently accounted for the overall separation after adjustment for multiple testing.

Adjusted analyses indicated that the probability of experiencing at least one postoperative complication varied across surgical techniques within this cohort, with extracapsular enucleation associated with the lowest proportion of affected patients. Patient-related factors also contributed to postoperative outcomes, as increased ΒΜΙ was associated with higher odds of postoperative complications, whereas no evidence of interaction between surgical technique and ΒΜΙ or alcohol consumption was observed.

### 4.1. Surgical Strategies in Contemporary Practice

Surgical management of benign parotid tumors encompasses a range of operative approaches that differ in extent of resection and underlying surgical philosophy [48,49,50]. Over recent decades, increasing attention has been directed toward tissue-preserving techniques, reflecting efforts to limit postoperative morbidity while maintaining functional outcomes [50,51]. Within this spectrum, extracapsular dissection has been adopted in selected centers for carefully chosen lesions, although its use remains variable and closely linked to institutional practice patterns and surgeon experience [52,53].

Most comparative data in the literature address extracapsular dissection in relation to more extensive procedures, particularly traditional superficial parotidectomy [11,54,55]. Interpretation of these comparisons is complicated by substantial heterogeneity across studies, including differences in patient selection, tumor characteristics, definitions of postoperative complications, and follow-up intensity [7,49]. As a result, reported morbidity rates often vary widely and may not be directly comparable across series. Favorable outcomes reported for extracapsular dissection frequently derive from cohorts enriched with superficially located or anatomically favorable tumors, whereas more complex lesions are more commonly managed with wider resections [52,55]. Consequently, existing evidence does not delineate a consistent gradient of postoperative morbidity across surgical techniques but instead reflects diverse clinical practices and selection processes.

Within this context, extracapsular enucleation has traditionally been approached cautiously, largely due to concerns regarding oncologic adequacy informed by historical experience with pleomorphic adenoma [48,50,56]. However, Warthin’s tumor constitutes a biologically distinct entity, typically characterized by encapsulation, non-infiltrative growth, and negligible malignant potential [57,58,59,60]. These features have prompted renewed consideration of extracapsular enucleation in selected cases. In a recent single-center validation study using predefined radiologic and cytologic criteria, 108 tumors managed with enucleation showed no cases of occult pleomorphic adenoma or malignancy and were associated with favorable perioperative outcomes, with the operative description indicating that the procedure was performed in an extracapsular plane [61].

### 4.2. Ιnterpretation of Postoperative Morbidity Findings

In this cohort, differences in postoperative morbidity among surgical techniques were observed primarily at the level of overall outcomes rather than being attributable to a single dominant complication. This observation suggests that morbidity may be more accurately characterized by the cumulative burden of postoperative events than by isolated complication-specific risks [62,63]. Additionally, even though extracapsular enucleation was associated with lower observed rates for most individual complications, none of these endpoints, when examined independently, was sufficient to explain the overall separation after adjustment for multiple comparisons.

This distinction is particularly relevant in benign parotid surgery, where individual complications tend to occur at relatively low frequencies and patient burden is more appropriately reflected by whether any postoperative morbidity occurs at all [63,64]. This approach is also consistent with the broader methodological literature on composite outcomes, which supports their use when clinically related events are individually infrequent but collectively reflect a meaningful patient-level burden, provided that the components are clearly defined and transparently reported [65,66]. Within this framework, absolute effect measures provide a pragmatic summary of the findings. In the present cohort, the adjusted probability of experiencing at least one postoperative complication was lower following extracapsular enucleation than after extracapsular dissection or partial superficial parotidectomy. The corresponding absolute risk differences indicate that selection of extracapsular enucleation instead of either comparator technique was associated with fewer patients experiencing postoperative morbidity during routine follow-up.

Nevertheless, this finding must be interpreted in the context of selective surgical decision-making, and the reported differences should be understood as reflecting outcomes achieved under specific anatomical and technical conditions rather than as evidence of an intrinsic risk profile independent of tumor characteristics or as an indication of procedural superiority. Additionally, even though overall morbidity provides the most informative summary outcome, examination of individual complication domains remains clinically important for contextual interpretation and for alignment with established surgical endpoints in benign parotid surgery.

Facial nerve morbidity represents the principal safety concern in this setting. In the present cohort, permanent facial nerve palsy was infrequent regardless of the surgical technique applied. This finding is consistent with previous reports indicating that the rate of postoperative facial palsy is relatively low [9,11,67,68]. The available literature further suggests that facial nerve risk is closely related to tumor location, depth, and the extent of dissection required, rather than to the surgical technique itself [69,70,71].

Frey’s syndrome constitutes another clinically relevant endpoint because of its impact on postoperative quality of life [8,72]. Prior studies have demonstrated lower rates following extracapsular dissection compared with superficial parotidectomy, with partial superficial parotidectomy showing intermediate outcomes [9,52,67,68]. In the present cohort, extracapsular enucleation was associated with a numerically lower incidence of Frey’s syndrome, a pattern compatible with preservation of parenchymal tissue planes and reduced disruption of autonomic nerve fibers, mechanisms thought to limit aberrant reinnervation [72,73].

Salivary fistula and sialocele—complications that may prolong recovery and necessitate additional interventions—were also less frequent following extracapsular enucleation. Reported rates after conservative parotid procedures vary widely in the literature, typically ranging from 2% to 10%, depending on definitions and surveillance intensity [9,52,67]. The lower incidence observed with extracapsular enucleation is biologically plausible and consistent with reports linking reduced dead space and limited parenchymal disruption to decreased postoperative salivary leakage [74]. Also, hematoma and seroma occurred at low and comparable frequencies across all surgical techniques, in agreement with existing evidence suggesting that these complications are influenced predominantly by perioperative hemostasis, patient comorbidity, and anticoagulant exposure rather than by the extent of glandular resection alone [75].

Finally, although surgical site infections were not predefined as a primary endpoint, they were considered clinically relevant and therefore examined in a contextual manner. In benign parotid surgery, even infrequent infectious complications may adversely affect postoperative recovery, prolong wound care, and necessitate additional medical interventions, thereby contributing meaningfully to the overall morbidity burden. In the present cohort, the observed pattern of surgical site infections was consistent with the broader morbidity profile across surgical techniques, with infections occurring more often in association with more extensive resections [76]. This observation aligns with previous reports indicating higher surgical site infections rates following procedures involving wider dissection fields and greater tissue disruption, such as partial superficial parotidectomy, compared with more conservative approaches [68,77,78]. From a mechanistic standpoint, the limited dissection, reduced dead space, and preservation of local tissue planes associated with extracapsular enucleation and extracapsular dissection provide a plausible explanation for a lower infectious risk, although comparative evidence remains limited [79].

### 4.3. Revisiting Oncologic Concerns Associated with Extracapsular Enucleation in Unifocal Warthin’s Tumor

Oncologic concerns toward extracapsular enucleation in parotid surgery have historically been shaped by experience derived from the pleomorphic adenoma literature, in which limited resections, and capsular violation or tumor spillage, were associated with increased recurrence risk [80]. Subsequent pathological and surgical investigations in pleomorphic adenoma have identified biological and technical features—such as pseudopodal extensions, satellite nodules, capsular irregularities, and susceptibility to intraoperative spillage—that provide a mechanistic rationale for recurrence following insufficient resection [56,81,82]. These observations contributed to a durable conceptual association between conservative surgical techniques and oncologic inadequacy in benign parotid surgery.

The applicability of this reasoning to other benign parotid tumors, however, warrants careful re-examination. In Warthin’s tumor, the well-documented propensity for multifocality and bilaterality complicates interpretation of postoperative “recurrence” as a purely surgical endpoint, as newly detected lesions may reflect intrinsic biological behavior rather than inadequate local excision [83,84,85,86]. Particularly in retrospective analyses, it often remains uncertain whether postoperative detection of additional lesions represents true failure at the surgical bed or metachronous tumor development consistent with the natural history of Warthin’s tumor.

Contemporary data in benign parotid surgery increasingly frames oncologic safety and surgical strategy around case selection rather than extent of resection alone. A meta-analysis demonstrated that even though extracapsular dissection had fewer complications when applied to carefully selected patients with well-defined, superficial, and anatomically favorable tumors, accompanied by reduced perioperative morbidity, it can yield recurrence rates comparable to superficial parotidectomy [7]. Therefore, the selection-focused approach reflects a shift away from traditional “maximal resection” concept toward a model where preoperative and intraoperative assessment of tumor characteristics informs the choice of technique. The current study’s findings are most appropriately interpreted as reflecting an institutional experience under defined diagnostic and intraoperative constraints, rather than as confirmatory evidence of long-term oncologic safety of extracapsular enucleation given that oncologic endpoints—such as local recurrence or metachronous disease—were not systematically assessed, particularly in the presence of limited follow-up and attrition.

### 4.4. Strengths and Limitations

This study has several methodological strengths that should be considered when interpreting its findings. First, the analysis was restricted to histologically confirmed, unifocal Warthin’s tumors, thereby excluding pleomorphic adenoma and multifocal disease and allowing disease-specific interpretation of postoperative morbidity. Second, the use of individual matching across clinically relevant patient-related variables (age, sex, smoking status, body mass index, and alcohol consumption) aimed to balance baseline susceptibility to postoperative complications rather than to reproduce surgical decision-making or eliminate confounding by indication [87,88].

In addition, all procedures were performed within a single institution by a surgical team that followed the same protocol, limiting variability in operative technique and perioperative management. Furthermore, the use of complementary analytical frameworks, including conditional logistic regression and alternative regression models yielding convergent estimates, further supports the internal consistency of the findings. Finally, the reporting of absolute risk differences and numbers needed to treat enhances clinical interpretability by translating relative associations into patient-level effect measures [89,90].

Yet several limitations must also be acknowledged. Despite matching and multivariable adjustment, surgical technique selection remained primarily driven by tumor-related anatomical characteristics, including size, depth, proximity to facial nerve branches, and local tissue conditions. These factors are central to operative planning but were not uniformly documented in the retrospective record and therefore could not be incorporated into the matching process or adjusted models, resulting in residual confounding by indication [91,92]. Accordingly, the lower morbidity observed with extracapsular enucleation may partly reflect more favorable anatomical profiles rather than an intrinsic procedural effect, and the findings should be interpreted within this context.

Additional limitations arise from the retrospective classification of surgical techniques. Although institutional protocols and operative reports were used to distinguish between extracapsular enucleation and extracapsular dissection, variability in documentation and terminology over the study period may have introduced misclassification in borderline cases [93]. Furthermore, the analytical cohort was restricted to cases without documented intraoperative capsular rupture or tumor spillage. A total of 16 such cases were excluded prior to matching (9 EN, 5 ECD, and 2 PSP). While this restriction allowed postoperative morbidity to be examined under technically uncomplicated conditions considered acceptable for conservative approaches, the uneven distribution of these exclusions suggests that technically more challenging EN cases may have been preferentially removed from the analytical cohort. Accordingly, the lower morbidity observed after EN should be interpreted within the context of carefully selected, technically uncomplicated resections and may underestimate morbidity in routine clinical practice across the full clinical spectrum of Warthin’s tumor [86,94].

Interpretation of absolute complication rates also warrants caution. Definitions of postoperative complications, particularly facial nerve dysfunction and Frey’s syndrome, vary substantially across published series. The comparison of the current study’s findings with other studies published in the literature should not be performed given that the present findings are most appropriately interpreted as reflecting an institutional experience under specific diagnostic and intraoperative constraints, rather than as confirmatory evidence of oncologic equivalence between surgical techniques.

The single-center design and temporal evolution of surgical practice during the study period constitute further limitations. Changes in institutional experience, case selection, and operative preference over time introduce the possibility of temporal and learning-curve effects, which cannot be fully excluded and may have contributed to the observed differences in morbidity. Consequently, the findings should be interpreted as descriptive of outcomes achieved within this institutional context rather than as evidence of causal superiority of one technique over another. Although the single-surgical-team design ensured technical consistency, it also limits external generalizability, as outcomes may differ in settings with different levels of expertise or perioperative protocols.

Issues related to data completeness must also be considered. Although no missing data were present for variables included in the final matched analytical cohort, this does not imply complete data availability across the entire source population. Patients with incomplete documentation for predefined matching variables or postoperative outcomes were excluded prior to matching. As a result, the analytical cohort represents a subset of patients with sufficiently detailed records, introducing potential selection bias related to data completeness, a limitation inherent to long-term retrospective studies relying on historical medical records [93].

Finally, the median follow-up duration may have been insufficient to capture late complications or metachronous tumor development, which may occur beyond five years after initial treatment [83,94]. Patient-reported outcome measures also were not available, despite their increasing relevance in benign parotid surgery [95]. Importantly, the present findings should not be interpreted as an endorsement of extracapsular enucleation or extracapsular dissection as universally applicable or technically trivial procedures. Both techniques require meticulous case selection, detailed anatomical knowledge, and substantial experience in facial nerve-preserving surgery, as application outside a structured and supervised setting may be associated with increased operative risk.

### 4.5. Implications for Future Research

The findings of the present study highlight several directions for future research aimed at refining the role of extracapsular enucleation and other conservative techniques in the management of unifocal Warthin’s tumor. Given the influence of tumor-related anatomical characteristics on surgical decision-making, future studies should prioritize prospective designs with systematic documentation of key anatomical variables, including tumor size, depth, relation to facial nerve branches, and local inflammatory changes [48,96]. Incorporation of such parameters would allow more granular stratification of risk and facilitate clearer separation between technique-related effects and selection-related factors.

Long-term oncologic outcomes remain another critical area requiring further investigation. Although Warthin’s tumor is biologically distinct from pleomorphic adenoma and carries negligible malignant potential, its known propensity for metachronous and bilateral disease necessitates extended follow-up [86,97]. Future prospective cohorts with standardized definitions of local recurrence versus metachronous tumor development, coupled with follow-up durations exceeding a decade, are needed to adequately characterize long-term disease behavior across surgical techniques.

Equally important is the integration of patient-reported outcome measures into future studies. While postoperative morbidity captures clinically relevant adverse events, it does not fully reflect patient experience in benign parotid disease. Quality of life, cosmetic satisfaction, sensory disturbances, and functional recovery are increasingly recognized as central outcomes in this setting and should be assessed using validated instruments in a standardized manner [98,99,100]. Such data would complement morbidity-based analyses and provide a more comprehensive evaluation of treatment impact.

Methodologically, multicenter collaboration represents a necessary step toward improving external validity. Although single-center studies ensure technical consistency, broader collaboration across institutions with varying expertise and practice patterns would enable evaluation of reproducibility and generalizability of findings. In this context, harmonization of complication definitions, follow-up protocols, and reporting standards would be essential to reduce heterogeneity and facilitate meaningful comparison across studies.

Finally, future research should also consider emerging minimally invasive and non-surgical approaches, including endoscopic and robotic-assisted parotidectomy and image-guided ablative techniques. While these modalities remain investigational in Warthin’s tumor, their potential role in carefully selected patients warrants systematic evaluation alongside established surgical strategies, particularly with respect to functional preservation and recovery trajectories.

## 5. Conclusions

In this single-center matched cohort limited to histologically confirmed, unifocal Warthin’s tumor, postoperative morbidity differed across surgical techniques, with extracapsular enucleation associated with lower short-term complication rates compared with extracapsular dissection and partial superficial parotidectomy.

Interpretation of these findings requires caution. Surgical technique selection was intrinsically linked to tumor-related anatomical features and intraoperative assessment, whereas matching addressed patient-related vulnerability to complications rather than determinants of surgical indication. Consequently, the observed differences should be understood as selection-dependent and context-specific, rather than as evidence of causal or inherent procedural superiority of extracapsular enucleation.

Clinically, the results support consideration of extracapsular enucleation only within a restricted indication framework, limited to unifocal, superficially located, well-encapsulated Warthin’s tumors with favorable anatomical relationships to the facial nerve. The data do not support extrapolation of this approach to more complex disease presentations, nor do they imply interchangeability with more extensive resections. Conservative techniques should not be interpreted as simplified procedures, but as operations that require advanced anatomical judgment and substantial surgical expertise.

Overall, these findings highlight the need to shift the discussion from comparisons of surgical extent toward more explicit documentation of selection criteria and tumor anatomy. Future multicenter studies with standardized definitions, prospective characterization of anatomical determinants, and extended follow-up are necessary to delineate the appropriate role of conservative surgical strategies in Warthin’s tumor and to contextualize morbidity outcomes within long-term disease behavior.

## Figures and Tables

**Table 1:** Baseline characteristics of the study population by surgical technique.

Characteristic, n (%)	ECD (n = 120)	PSP (n = 120)	EN (n = 120)	*p*-Value *
**Sex**				
Male	66 (55.0)	66 (55.0)	66 (55.0)	
Female	54 (45.0)	54 (45.0)	54 (45.0)	
**Age group**				
<40 years of age	2 (1.7)	2 (1.7)	2 (1.7)	
40–49 years of age	35 (29.2)	35 (29.2)	35 (29.2)	
50–59 years of age	36 (30.0)	36 (30.0)	36 (30.0)	
60–69 years of age	31 (25.8)	31 (25.8)	31 (25.8)	
≥70 years of age	16 (13.3)	16 (13.3)	16 (13.3)	
**Smoking status**				0.914
No	11 (9.2)	9 (7.5)	13 (10.8)	
Former	13 (10.8)	13 (10.8)	11 (9.2)	
Active	96 (80.0)	98 (81.7)	96 (80.0)	
**BMI category**				0.053
Normal	35 (29.2)	32 (26.7)	46 (38.3)	
Overweight	40 (33.3)	51 (42.5)	48 (40.0)	
Obese	45 (37.5)	37 (30.8)	26 (21.7)	
**Alcohol use**				0.038
No	39 (32.5)	28 (23.3)	39 (32.5)	
Occasional	53 (44.2)	42 (35.0)	44 (36.7)	
Frequent	28 (23.3)	50 (41.7)	37 (30.8)	

* Pearson’s χ^2^ or Fisher’s exact test for *p*-values. Standardized mean differences (SMDs) for BMI: Normal = 0.11, Overweight = 0.08, Obese = 0.14; for Alcohol use: None = 0.09, Occasional = 0.08, Frequent = 0.16; for Smoking status: No = 0.058, Former = 0.037, Active = 0.028. Values <0.1 were considered negligible. Small residual imbalance in partially matched variables was addressed in adjusted conditional logistic regression and risk-standardization models.

**Table 2:** Unadjusted postoperative complication rates by surgical technique.

Complication	ECD n (%)	PSP n (%)	EN n (%)	*p*-Value *
**Overall complications**	35 (29.2)	39 (32.5)	10 (8.3)	<0.001 ^†^
Frey’s syndrome	5 (4.2)	7 (5.8)	1 (0.8)	0.093 ^‡^
Salivary fistula	8 (6.7)	8 (6.7)	1 (0.8)	0.035 ^†^
Seroma	13 (10.8)	10 (8.3)	4 (3.3)	0.071 ^†^
Hematoma	9 (7.5)	12 (10.0)	3 (2.5)	0.052 ^†^
Permanent GAN dysfunction	6 (5.0)	8 (6.7)	1 (0.8)	0.055 ^‡^
Permanent facial nerve palsy	2 (1.7)	6 (5.0)	0 (0.0)	0.031 ^‡^

* Pearson’s χ^2^ or Fisher’s exact test for *p*-values (^†^ Pearson’s χ^2^ test, ^‡^ Fisher’s exact test). Severe morbidity was defined as hematoma requiring re-exploration and salivary fistula or sialocele necessitating repeated interventions.

**Table 3:** Relative effect estimates for overall postoperative complications. Odds ratios were estimated using conditional logistic regression stratified by matched triplet. Extracapsular dissection was used as the reference category for surgical technique. The comparison between extracapsular enucleation and partial superficial parotidectomy was derived from post-estimation contrasts. Models were adjusted for smoking status, body mass index, alcohol consumption, sex, and age group.

Comparison	OR ^1^	95% CI ^2^	*p*-Value
**EN ^†^ vs. ECD ^‡^**	0.23	0.10–0.50	<0.001
PSP * vs. ECD ^‡^	1.24	0.68–2.28	0.482
EN ^†^ vs. PSP *	0.18	0.08–0.41	<0.001

^1^ Odds Ratio; ^2^ Confidence Interval; ^†^ Extracapsular enucleation; ^‡^ Extracapsular dissection; * Partial superficial parotidectomy.

**Table 4:** Covariate-adjusted absolute risks and clinical effect measures for overall postoperative complications.

Comparison ^1^	Risk of Complication (%) ^2^	Absolute Risk Difference (95% CI) ^3^	NNT ^4^
**EN ^†^**	8.3	Ref. ^§^	–
**ECD ^‡^ vs. EN ^†^**	29.3	+21% (11–31)	4.8 (3.2–8.8)
PSP * vs. EN ^†^	32.4	+24% (14–34)	4.2 (2.9–7.1)

^1^ **Comparison:** Pairwise comparison between surgical techniques, with enucleation used as the reference category; ^2^ **Adjusted risk of complication (%):** Covariate-adjusted probability of experiencing at least one postoperative complication, estimated from logistic regression models including surgical technique, smoking status, body mass index, alcohol consumption, sex, and age group; ^3^ **Absolute risk difference vs. enucleation (95% CI):** Absolute difference in the adjusted probability of postoperative complications between each surgical technique and enucleation. Confidence intervals were estimated using nonparametric cluster bootstrap resampling at the matched triplet level (2000 replicates); ^4^ **Number needed to treat (NNT):** Number of patients who would need to be treated with enucleation instead of the comparator surgical technique to prevent one additional postoperative complication. NNTs were calculated as the inverse of the corresponding absolute risk differences; ^†^ Extracapsular enucleation; ^‡^ Extracapsular dissection; * Partial superficial parotidectomy; ^§^ Reference.

## Data Availability

All data analyzed in this study are available from the corresponding author upon reasonable request.

## Supplementary Materials

The following supporting information can be downloaded at: https://doi.org/10.5281/zenodo.19153727, Figure S1. Flow diagram of study selection, exclusions, and 1:1:1 matching process.
